# Increased prothrombotic property as a risk factor of acute kidney injury after surgical repair of abdominal aortic aneurysm: a prospective observational study

**DOI:** 10.1186/s40560-014-0046-3

**Published:** 2014-07-16

**Authors:** Yasushi Innami, Nobuyuki Katori, Katsuya Mori, Shizuko Kosugi, Takeshi Suzuki, Norihiro Sakurai, Hiromasa Nagata, Junzo Takeda, Hiroshi Morisaki

**Affiliations:** 1 Department of Anesthesiology, Keio University School of Medicine, 35 Shinanomachi, Shinjuku 160-8582, Tokyo, Japan

**Keywords:** ADAMTS13, Acute kidney injury, AKI, Neutrophil gelatinase-associated lipocalin, NGAL, Abdominal aortic aneurysm

## Abstract

**Background:**

Acute kidney injury (AKI) is one of the major morbidities after surgical repair of abdominal aortic aneurysm (AAA); however, precise pathogenesis of this morbidity has not been well determined. Since prothrombotic coagulation abnormality may precede organ dysfunction in systemic inflammatory state, we examined the kinetics of von Willebrand factor (VWF) and a disintegrin-like metalloprotease with thrombospondin type 1 motif 13 (ADAMTS13), a cleaving enzyme of VWF, on the development of AKI after AAA surgery.

**Methods:**

The kinetics of ADAMTS13 and VWF were examined in ten patients who underwent surgical repair of AAA. The changes in plasma neutrophil gelatinase-associated lipocalin (NGAL), a novel biomarker for AKI, and serum creatinine concentration were also examined at four points until seventh postoperative day (POD). Clinical diagnosis of AKI was based on the change in serum creatinine concentration and urine output according to Acute Kidney Injury Network (AKIN) criteria.

**Results:**

ADAMTS13 activity was significantly lower than normal level before the surgery and showed a trend of decrease toward 3POD. The VWF/ADAMTS13 ratio showed a significant increase on 1POD, which persisted until 7POD. None of patents was diagnosed as AKI based on AKIN criteria, although two patients received furosemide and/or carperitide therapy because of decreased urine output less than 0.5 ml/kg/h for several hours in ICU. Plasma NGAL showed a trend to increase after the surgery, which was significant on 3POD. The change in plasma NGAL was significantly correlated with VWF/ADAMTS13 ratio (*P* < 0.01).

**Conclusions:**

This study has shown that patients undergoing AAA surgery were prothrombotic after the surgery because of high VWF/ADAMTS13 ratio. Correlation between VWF/ADAMTS13 ratio and NGAL might indicate contribution of thrombotic event to subclinical AKI in the patients undergoing AAA surgery.

## Background

Acute kidney injury (AKI) is one of the major morbidities after surgical repair of abdominal aortic aneurysm (AAA). The incidence of AKI after AAA surgery, although it depends on the definition of AKI, varies between 22% and 75%, which is extremely high in ruptured AAA [1]-[3]. The mechanisms to develop perioperative AKI include atherosclerosis of renal artery, renal ischemia-reperfusion injury due to aortic cross-clamp, circulatory failure, and nephrotoxic agents as well as the neuroendocrine and inflammatory stress response to the surgery [4]-[6].

Recent investigations indicated that deterioration of the balance between von Willebrand factor (VWF) and its cleaving protease was associated with organ dysfunction including AKI in critically ill patients [7],[8]. Von Willebrand factor, which is an essential glycoprotein in platelet adhesion and aggregation at the vascular injury site, is synthesized in endothelial cells and megakaryocytes and is released into the blood stream by stimulation of inflammatory mediators such as interleukins or tumor necrosis factor α [9]. VWF is released from endothelial cells in the form of unusually large VWF multimer (UL-VWF) which can induce abnormal platelet aggregation and thrombus formation [10]. This abnormal thrombus formation due to UL-VWF could cause thrombotic microangiopathy (TMA), which results in organ dysfunction. But thrombogenic potential of UL-VWF is usually regulated by a protease secreted from hepatic stellate [10], named ADAMTS13 (a disintegrin-like metalloprotease with thrombospondin type 1 motif 13). ADAMTS13 cleaves UL-VWF into smaller multimers and reduces agglutinative properties of UL-VWF. Hence, the decrease in ADAMTS13 activity is related to the increase in circulating UL-VWF and plasma VWF antigen level, which may contribute to organ dysfunction subsequent to TMA [11],[12]. As ADAMTS13 activity is attenuated by systemic inflammation [12], decrease in ADAMTS13 activity induced by inflammatory cytokines in major aortic surgery could contribute to the development of postoperative organ dysfunction including AKI.

Few studies to date, however, have examined the change in ADAMTS13 activity in AAA surgery or the contribution of the balance between ADAMTS13 and VWF to development of prothrombotic coagulation abnormalities. Hence, we conducted a prospective observational study to investigate perioperative change in ADAMTS13 activity and also a relationship between prothrombotic property indicated as VWF antigen/ADAMTS13 activity ratio and the development of AKI in patients undergoing AAA surgery.

## Methods

### Patients

With approval of Institutional Review Board of Keio University Hospital (Tokyo, Japan) and written informed consents, adult patients undergoing elective open infrarenal abdominal aortic surgery were included in the study. The exclusion criteria for the study were preexisting renal failure requiring hemodialysis and/or preoperative serum creatinine concentration (Cr) ≥2 mg/dl, active hepatitis or cirrhosis, and hemostatic disorder. Postoperative AKI was defined by AKIN criteria: the Cr increased by more than 0.3 mg/dl compared with the preoperative value; the percent increase in Cr was more than 50% compared with the preoperative value; urine output less than 0.5 ml/kg/h continued for more than 6 h [13].

### Anesthesia management

After the placement of an epidural catheter at Th7/8 or 8/9 level, general anesthesia was induced with propofol and rocuronium, and the trachea was intubated. Anesthesia was maintained with sevoflurane (1.5%−2.5%) in 40%−60% oxygen and thoracic epidural analgesia. Mechanical ventilation was performed to maintain arterial carbon dioxide pressure between 30 and 40 mmHg. All patients received a radial arterial catheter with FloTrac™ sensor (Edwards Lifesciences, CA, USA) and PreSep™ oximetry catheter (Edwards Lifesciences, CA, USA) via internal jugular vein as hemodynamic monitors in conjunction with routine monitors including electrocardiogram and pulse oximeter. Fluid therapy was managed with the infusions of crystalloid and hydroxyethyl starch, guided by mean arterial blood pressure, cardiac output, and central venous pressure, and central venous oxygen saturation. Erythrocyte salvage device (Cell Saver®, Haemonetics Japan GK, Tokyo, Japan) was applied in all cases to salvage red blood cells during the surgery. Transfusion of blood products including irradiated packed red blood cells (RBC), fresh frozen plasma (FFP), or platelet concentrate (PC) was decided by attending anesthesiologists according to the results of complete blood count or coagulation tests. Vasoactive agents including phenylephrine or ephedrine were administered intermittently to maintain mean arterial blood pressure more than 60 mmHg to obviate the risk of perioperative AKI [3]. Routine use of diuretic agents including furosemide, low-dose dopamine, or carperitide was avoided, whereas all patients received the infusion of 1 g/kg D-mannitol before aortic cross-clamp as an institutional protocol. All patients received 100 unit/kg of unfractionated heparin as a primary dose for anticoagulation during aortic cross-clamp. After the confirmation that celite-activated clotting time (ACT) reached more than 250 s, the infrarenal aorta was cross-clamped. ACT was monitored every 30 min and additional heparin was administered to maintain ACT between 200 and 300 s if needed until all vascular anastomosis was finished. Minimum required dose of protamine was administered if ACT was prolonged more than 200 s after the reperfusion. The trachea was extubated in all patients in the operation room, and they were transferred to the ICU.

### Sample collection

Twenty milliliters of blood was collected from the patients at the following four time points: (1) preoperative, (2) the first postoperative day (1POD), (3) 3POD, and (4) 7POD. Twelve milliliters of the blood was immediately sent to the laboratory for complete blood count, Cr, and coagulation tests as a routine clinical examination in AAA surgery. Eight milliliters of the blood was collected into citrate tubes and immediately centrifuged at 1,500×*g* for 10 min, and the plasma was stored at −80°C for the assays of ADAMTS13 activity, VWF antigen, cytokines, and neutrophil gelatinase-associated lipocalin (NGAL). Activity of ADAMTS13 was assayed by commercial enzyme-linked immunosorbent assay (ELISA) kit (Kainos Laboratories Inc., Tokyo, Japan). Plasma VWF antigen was measured using rabbit antihuman VWF polyclonal antibody (Dako, Kyoto, Japan). As an appearance of UL-VWF in plasma has been demonstrated in patients with inadequate function or decreased level of ADAMTS13 [11],[12], plasma UL-VWF should be a marker to indicate the imbalance between ADAMTS13 activity and VWF. But UL-VWF assay, which is usually performed by electrophoresis analysis, is unfeasible for real-time diagnosis or therapeutically monitoring. VWF functional assays such as collagen-binding activity or ristocetin cofactor activity are conventional to determine activity of VWF multimer pattern; however, recent investigations indicated the change in VWF antigen/ADAMTS13 ratio rather than functional VWF assays could be relate to thrombotic complications or organ dysfunction [8],[14],[15]. Thus, we adopted VWF antigen assay to determine VWF/ADAMTS13 ratio. Both ADAMTS13 activity and VWF antigen level are expressed as a percentage of normal control plasma.

Three cytokines including interleukin-6 (IL-6), IL-8/CXCL8, and tumor necrosis factor α (TNF-α) were measured in duplicate by ELISA using commercially available kit (Procarta cytokine Assay kit, Affymetrix Inc., CA, USA). NGAL is a novel biomarker for AKI, which has high sensitivity and specificity in detection of AKI [16]. NGAL was measured in duplicate using commercially available ELISA kit (Human Lipocalon-2/NGAL ELISA kit, R&D systems Inc., MN, USA).

### Statistical analysis

Data were expressed as mean ± standard deviation unless otherwise specified. A commercial statistical software package (SigmaPlot 12; Systat Software Inc., CA, USA) was used to analyze the data. Kruskal-Wallis H-test, followed by Mann-Whitney *U* test with Bonferroni correction, was used to compare the serial data in a time course. Spearman correlation coefficient was calculated to determine the relationship between two parameters where applicable. Two-tailed *P* value less than 0.05 was considered significant.

## Results

Fourteen patients were included in the study. Four of 14 patients were excluded because of the lack of ADAMTS13 and VWF data. Patient characteristics are presented in Table 1. Three patients had serum creatinine concentration more than 1.5 mg/dl, and two of them received the medication of oral furosemide. Six patients received antiplatelet therapy with aspirin (*n* = 4) or aspirin + clopidogrel (*n* = 2), but these antiplatelet drugs were withdrawn 10–14days prior to the surgery. None of the patients received anticoagulation therapy before the surgery. Table 2 indicates results of preoperative coagulation and fibrinolysis examination. Mean plasma concentrations of fibrin/fibrinogen degradation products and D-dimer were higher than normal level, which indicated increased plasmin activity in preoperative period. Table 3 shows intraoperative and postoperative variables. Two patients received furosemide during anesthesia due to transient decrease in urine output despite the appropriate fluid therapy and hemodynamic parameters. Two patients received a continuous infusion of dopamine due to persistent hypotension (mean arterial blood pressure < 60 mmHg despite a total dose of phenylephrine 1 mg or ephedrine 40 mg). No patients received either FFP or PC during the study periods.

**Table 1:** Patient characteristics

**Patient characteristics**	**Values**
Gender (male/female)	8/2
Age (years)	71 ± 10
Height (cm)	164 ± 9
Weight (kg)	62 ± 14
Diabetes	7 (70%)
Hypertension	10 (100%)
CAD	4 (40%)
Atrial fibrillation	2 (20%)
sCr ≥ 1.5 mg/dl	3 (30%)
Medication	
Calcium antagonist	6 (60%)
ACE inhibitor	3 (30%)
ARB	2 (20%)
β-blocker	3 (30%)
Furosemide	2 (20%)
Antiplatelets	6 (60%)
Aspirin 4, Aspirin + Clopidogrel 2	

*CAD* coronary artery disease, *sCr* serum creatinine, *ACE inhibitor* angiotensin converting enzyme inhibitor, *ARB* angiotensin receptor blocker.

**Table 2:** Preoperative coagulation and fibrinolysis examination

	**Mean ± SD**	**Normal range**
Hemoglobin	13.4 ± 1.2 (g/dl)	
	F, 12.0 ± 0.6 (g/dl)	12.5–15.0 g/dl
	M, 13.7 ± 1.0 (g/dl)	13.5–17.0 g/dl
Platelet count	190 ± 98 (×10^3^/μl)	160–350 × 10^3^/μl
APTT	31.1 ± 1.5 (s)	23–36 s
PT-INR	1.07 ± 0.09	0.8–1.2
Fibrinogen	320 ± 47 (mg/dl)	160–350 mg/dl
FDP	6.7 ± 4.4 (μg/ml)	<5 μg/ml
D-dimer	4.2 ± 3.4 (μg/ml)	<1 μg/ml

*F* and *M* female and male, respectively, *APTT* activated prothrombin time, *PT-INR* international normalized ratio/prothrombin time, *FDP* fibrin/fibrinogen degradation products.

**Table 3:** Intraoperative and postoperative variables

**Variables**	**Values**
Intraoperative variables	
Anesthesia time (min)	339 ± 62
Operation time (min)	236 ± 47
Aortic cross-clamp time (min)	58 ± 18
Blood loss (ml/kg)	20.1 ± 12.4
Urine (ml · kg^−1^ · hr^−1^)	3.3 ± 2.1
Infusion (ml · kg^−1^ · hr^−1^)	13.0 ± 5.1
Blood transfusion	
Salvaged red blood cells (ml/kg)	8.9 ± 6.2
RBC (ml/kg)	5.6 ± 6.1
Furosemide	2 (20%)
Dopamine	2 (20%)
Postoperative variables	
Medication in ICU	
Furosemide	2 (20%)
Dopamine	3 (30%)
Carperitide	1 (10%)
Hemodialysis in 30 days	0 (0%)

*RBC* irradiated packed red blood cells.

**Figure 1:**
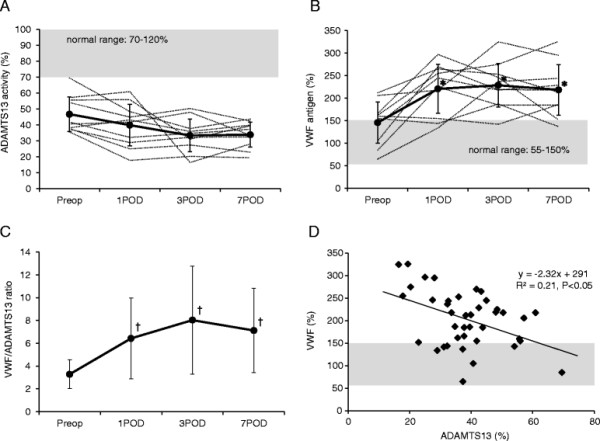
**Change in ADAMTS13 activity, VWF antigen, VWF/ADAMTS13 ratio, and correlation between ADAMTS13 and VWF. (A)** Change in ADAMTS13 activity. **(B)** Change in VWF antigen. **(C)** Change in VWF/ADAMTS13 ratio. **(D)** Correlation between ADAMTS13 activity and VWF antigen. Solid lines represent mean values (SD), and dotted lines represent the native values. Shaded areas represent the normal ranges. *P < 0.01 vs. preoperative value. †*P* < 0.05 vs. preoperative value. *Preop* preoperative. ADAMTS13 activity was lower than the normal range through the study period and showed a trend of decrease after the surgery. VWF antigen increased significantly after the surgery. There was a significant increase in VWF/ADAMTS13 ratio after the surgery (*P* < 0.05). There was a significant inverse correlation between ADAMTS13 activity and VWF antigen (*P* < 0.05).

Figure 1 illustrates the changes in ADAMTS13 activity, VWF antigen, VWF/ADAMTS13 ratio, and correlation between ADAMTS13 activity and VWF antigen during the study periods. Preoperative values of ADAMST13 were lower than the normal range in all patients. ADAMTS13 showed a trend of decrease after the surgery, but it was not statistically significant (Figure 1A). VWF antigen increased significantly after the surgery and maintained a supernormal level in the study period (*P* < 0.01) (Figure 1B). Similar to the trend of VWF, VWF/ADAMTS13 ratio presented a significant increase after the surgery (*P* < 0.05) (Figure 1C). There was a significant inverse correlation between ADAMTS13 and VWF (*R*^2^ = 0.21, *P* < 0.05) (Figure 1D).

**Figure 2:**
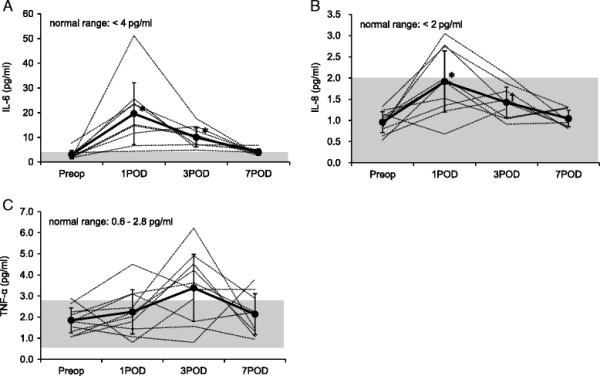
**Change in IL-6, IL-8, and TNF-α. (A)** Change in plasma IL-6. **(B)** Change in plasma IL-8. **(C)** Change in plasma TNF-α. Solid lines represent mean values (SD), and dotted lines represent the native values. Shaded areas represent the normal ranges of the plasma cytokine concentration. *P < 0.01 vs. preoperative value. †*P* < 0.05 vs. preoperative value. IL-6 and IL-8 increased significantly on 3POD and 7POD; however, IL-8 changed within the normal range. There was no significant change in TNF-α.

Figure 2 illustrates the change in IL-6, IL-8, and TNF-α. IL-6 showed clinically and statistically significant increase on 1POD (*P* < 0.01) and started to decrease towards 7POD (Figure 2A). The change in IL-8 showed a similar trend to IL-6 although it remained within the normal range. TNF-α showed a trend of increase towards 3POD although it was not statistically significant. Both IL-6 and IL-8 had significant but weak correlations with VWF/ADAMTS13 ratio (*R*^2^ = 0.12, *P* = 6.13 × 10^−5^ and *R*^2^ = 0.04, *P* = 0.004, respectively), whereas there was no correlation between TNF-α and VWF/ADAMTS13 ratio.

**Figure 3:**
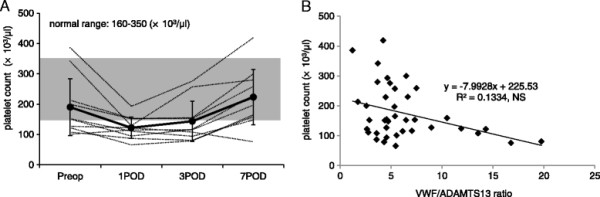
**Change in platelet count and correlation between VWF/ADAMTS13 ratio and platelet count. (A)** Change in platelet count. **(B)** Correlation between VWF/ADAMTS13 ratio and platelet count. Shaded areas represent the normal range of platelet count. Platelet count decreased towards 1POD (not significant) and showed a trend of recovery towards 7POD. Platelet count demonstrated a trend of decrease in coordination with the increase in VWF/ADAMTS13 ratio, although it was not significant.

Figure 3 illustrates the change in platelet count and the correlation between VWF/ADAMTS13 ratio and platelet count. Platelet count reached a nadir level on 1POD (not significant) and showed a trend of increase towards 7POD. Platelet count demonstrated the trend of decrease in coordination with the increase in VWF/ADAMTS13 ratio although it was not significant.

None of the patients had significant increase in Cr postoperatively. Two patients, who had baseline Cr values more than 1.5 mg/dl, received continuous infusion of carperitide and/or IV furosemide because their urine output decreased less than 0.5 ml/kg/h continuing for several hours on 3POD and 4POD, respectively. They received intermittent IV furosemide for the next 3 days and maintained their urine output. Including these two patients, none of the patients had persistent decrease in urine output throughout the study periods. Hence, there was no patient who was diagnosed as AKI according to AKIN criteria.

**Figure 4:**
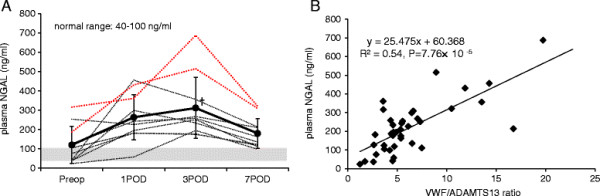
**Change in NGAL, and correlation between VWF/ADAMTS13 ration and NGAL. (A)** Change in plasma NGAL. Solid line represents mean values (SD), and dotted lines represent the native values. The dotted lines in red represent the values of the patients who received furosemide therapy. Shaded area represents the normal range of plasma NGAL. †*P* < 0.05 vs. preoperative value. Plasma NGAL increased significantly on 3POD. **(B)** Correlation between VWF/ADAMTS13 ration and plasma NGAL. There was a significant correlation between VWF/ADAMTS13 ratio and plasma NGAL (*P* < 0.01).

Figure 4 illustrates the changes in plasma NGAL and the correlation between VWF/ADAMTS13 ratio and plasma NGAL. Plasma NGAL showed a trend to increase after the surgery, which was significant on 3POD (Figure 4A). The patients who received IV furosemide had their maximum plasma NGAL values of 515 and 687 ng/ml on 3POD, respectively. The change in plasma NGAL was correlated with VWF/ADAMTS13 ratio significantly (*R*^2^ = 0.54, *P* = 7.76 × 10^−5^) (Figure 4B).

## Discussion

The present study showed that the ratio of VWF/ADAMTS13 increased significantly after the AAA surgery, which may indicate increasing risk of prothrombotic coagulation disorder. It also showed significant correlation between inflammatory cytokines including IL-6 and IL-8 and VWF/ADAMTS13 ratio. Besides, NGAL value which increased significantly after the surgery was correlated with VWF/ADAMTS13 ratio. Collectively, the systemic inflammation was associated with the increase in the ratio of VWF/ADAMTS13 after the AAA surgery. Simultaneously, the increased VWF/ADAMTS13 ratio, indicating increased risk of thrombosis, was associated with the increase in plasma NGAL, one of the most sensitive biomarkers for AKI [16].

Several investigations demonstrated perioperative change in ADAMTS13 activity [17]-[19], whereas few studies have been performed in major aortic surgery. The current study demonstrated that patients with AAA had significantly lower ADAMST13 activity than those undergoing cardiac surgery in preoperative period [18],[19]. Aortic aneurysm is associated with change of coagulation system characterized by increased thrombin generation and subsequent plasmin production, which is proportional to the volume of intraluminal thrombus [20]. Since preoperative acceleration of fibrinolysis was recognized in the current study, degradation of ADAMTS13 by thrombin and/or plasmin could partly explain the decreased level of ADAMTS13 activity in patients with aortic aneurysm [21]. Several mechanisms to account for the acute decline of ADAMTS13 have been postulated such as accelerated consumption, suppressed production or secretion [22], and increased VWF antigen. Previous observations indicated the increase in plasma VWF including UL-WVF, no matter which is endogenous or exogenous, could induce decrease in ADAMTS13 activity [23],[24]. Several interleukins are also postulated to affect ADAMTS13 activity in patients with systemic inflammation [25],[26]. IL-6 showed a marked increase after the surgery in correlation with VWF/ADAMTS13 ratio in the current study. In addition to the systemic inflammatory response to surgical insults, the direct release of IL-6 from aortic aneurysm might contribute to the increase in IL-6 after AAA surgery [27]. Increased levels of inflammatory cytokines induced by surgical insults could be one of the reasons for decreased ADAMTS13 activity because inflammatory cytokines including IL-6 and IL-8 enhances the release of UL-VWF from endothelial cells and simultaneously inhibits cleavage of UL-VWF by ADAMTS13 [26]. Although the mechanisms that explain decrease in ADAMTS13 activity is multifactorial, inverse or mirror image pattern between VWF antigen level and ADAMTS13 activity observed in the current was consistent with the results in the previous studies [23],[24].

The most important finding in the current study was significant correlation between VWF/ADAMTS13 ratio and plasma NGAL which is a novel biomarker for AKI. In the setting of acute tubular injury, NGAL undergoes rapid and profound upregulation with large increase in both urine and plasma [28],[29]. In the current study, urine output decreased in two patients who had high NGAL values after the surgery. And these two patients received furosemide and/or carperitide therapy. As a result, an early treatment according to clinical decision might obviate the development of AKI. Since one of the clinical manifestations of TTP is kidney injury, which results from systemic TMA induced by severe deficiency of ADAMTS13 with high VWF antigen/ADAMTS13 ratio [30], the correlation between VWF/ADAMTS13 ratio and plasma NGAL may cast light on the elucidation of pathogenesis of AKI after AAA surgery.

Recent data provide evidence that an altered ADAMTS13 activity and a subsequent shift in the multimeric pattern of VWF may contribute to thrombocytopenia and microcirculatory failure due to TMA, which results in organ dysfunction including AKI [8],[9],[31]. Several investigators postulated that ADAMTS13 deficiency was associated with AKI in patients with sepsis [7]. Martin et al. also demonstrated that 50% of patients with severe sepsis suffered from AKI in whom the median ADAMTS13 activity was 43.2% [26]. This value is similar to the result in the current study, whereas none of the patients was diagnosed as AKI based on AKIN criteria. Such discrepancy between sepsis and AAA patients might be attributed to the difference in severity and persistence of systemic inflammatory response. Thrombocytopenia due to abnormal platelet aggregation is usually recognized in patients with high VWF/ADAMTS13 ratio. We found a trend of decrease in platelet count with higher VWF/ADAMTS13 ratio in the current study although it was not significant. Because bleeding during the surgery also decreases platelet count, it might be difficult to prove statistically significant correlation between VWF/ADAMTS13 ratio and platelet count in patients undergoing major aortic surgery. Furthermore, decrease in platelet count due to bleeding during the surgery might mask clinical manifestation or development of TMA in spite of high VWF/ADAMTS13 ratio.

There are several limitations to interpret the data herein. First, this study enrolled 14 patients and only 10 patients were accepted for the statistical analysis. We could not exclude the contribution of this small sample size on the detection of AKI. We estimated the incidence of AKI after AAA surgery as 40% and calculated the sample size to show statistical significance as 24 cases when we made the study protocol. However, we had to cease the study after including 14 cases because the elective open AAA surgery was almost replaced by endovascular surgery in our institute. Second, we did not directly measure UL-VWF level which is the most important factor that induces abnormal platelet aggregation. An ideal method was to examine both VWF antigen level and distribution of VWF multimers using electrophoresis analysis to prove existence of UL-VWF as shown in previous studies [7],[32]; however, electrophoresis analysis is not always feasible in clinical diagnosis and therapeutic monitoring. VWF activity assays are also useful to evaluate contribution of VWF to platelet adhesion or aggregation. Claus et al. investigated qualitative and quantitative variations of VWF and ADAMTS13 in patients with inflammation of varying severity, but they could not find a significant correlation between VWF activity and TMA or severity of organ dysfunction [8]. They concluded that VWF antigen/ADAMTS13 ratio could be a helpful parameter to identify patients with high risk of TMA or organ dysfunction due to systemic inflammation. Recent investigations also indicated that high VWF antigen level accompanied by decreased ADAMTS13 activity (high VWF antigen/ADAMTS13 ratio) could be a good parameter to evaluate prothrombotic properties or severity of diseases [15],[33],[34]. Third, the current study could not demonstrate the direct link between high VWF/ADAMTS13 ratio and TMA in the kidney. We did not examine histological change in the kidney because it is too invasive in the clinical setting although Bockmeyer et al. demonstrated that TMA induced by the ADAMTS13 deficiency was associated with the development of tubular injury and consequent AKI in a porcine model [35].

## Conclusions

The current study demonstrated that increased VWF/ADAMTS13 ratio was associated with the increase in IL-6 which reflects systemic inflammation in AAA surgery. Furthermore, there was significant correlation between VWF/ADAMTS13 ratio and NGAL which is a sensitive early phase biomarker reflecting renal tubular injury. As the association between high VWF/ADAMTS13 and TMA has been indicated, prothrombotic coagulation abnormality due to systemic inflammation might be one of the contributions to the development of AKI after AAA surgery. Further study is needed to confirm our findings.

## Abbreviations

AAA: abdominal aortic aneurysm

ACT: activated clotting time

ADAMTS13: a disintegrin-like metalloprotease with thrombospondin type 1 motif 13

AKI: acute kidney injury

NGAL: neutrophil gelatinase-associated lipocalin

TMA: thrombotic microangiopathy

UL-VWF: unusually large von Willebrand factor

VWF: von Willebrand factor

## Competing interests

The authors declare that they have no competing interests.

## Authors' contributions

YI participated in the analysis of the data and drafted the manuscript. NK designed the study protocol and conducted the study. KM participated in assay of NGAL and ADAMTS13. SK participated in the analysis and interpretation of the data. TS helped in the assay and analysis of the data. NS and HN helped in the analysis of the data. JT participated in the conduction of the study. HM participated in its design and coordination and helped to draft the manuscript. All authors read and approved the final manuscript.
